# P01.45. Activated neuropathway from nucleus tractus solitarius to rostal ventrolateral medulla during electroacupuncture

**DOI:** 10.1186/1472-6882-12-S1-P45

**Published:** 2012-06-12

**Authors:** Z Guo, J Longhurst

**Affiliations:** 1 University of California, Irvine, Irvine, USA

## Purpose

Our previous studies have shown that electroacupuncture (EA) at the Jianshi-Neiguan acupoints (P5-P6, overlying the median nerve) attenuates sympathoexcitatory responses through its influence on neuronal activity in the rostral ventrolateral medulla (rVLM). Nucleus tractus solitarius (NTS) receives inputs from somatic nerve stimulation. However, there is no information on the activation of NTS neurons by EA at P5-P6 acupoints, which subsequently affects the rVLM. Thus, the present study evaluated neuronal activation of NTS in response to EA, with regard to their projections to the rVLM.

## Methods

Seven to ten days after unilateral microinjection of a rodamine-conjugated microsphere retrograde tracer (100 nl) into the rVLM, rats were subjected to EA or served as a sham-operated control. EA was performed for 30 min at P5-P6 acupoints bilaterally.

## Results

Perikarya containing the microsphere tracer were found in the NTS of both groups. Compared to controls (needle placement without electrical stimulation, n=4), c-Fos immunoreactivity and neurons double-labeled with c-Fos, an immediate early gene and the tracer were more frequently found in the NTS of EA-treated rats (n=5), particularly, in the medial and lateral subdivisions of caudal and intermediate NTS extension.

## Conclusion

These results suggest that EA at P5-P6 acupoints activates NTS neurons. Furthermore, EA-activated NTS neuron can directly project to the rVLM, which is known to participate in EA-modulation of sympathetic activity.

